# The mediating role of testosterone in the relationship between body fat percentage and diabetes mellitus risk

**DOI:** 10.3389/fendo.2026.1872434

**Published:** 2026-07-14

**Authors:** Dongdong Wang, Congcong Jiao

**Affiliations:** 1 Department of Nephrology, The Fourth Affiliated Hospital of Nanjing Medical University (Nanjing Pukou Hospital), Nanjing, China; 2 Department of Nephrology, Shengjing Hospital of China Medical University, Shenyang, China

**Keywords:** body fat percentage, cross-sectional study, diabetes mellitus, mediation analysis, Mendelian randomization, testosterone

## Abstract

**Background:**

Obesity is one of the major risk factors for diabetes mellitus (DM), and testosterone plays a pivotal role in metabolic regulation. However, it remains unclear whether testosterone exerts a mediating effect in the association between body fat percentage (BFP) and DM.

**Methods:**

Clinical data (N = 4433) from Shengjing Hospital were used to assess the BFP-DM association, modeled via restricted cubic spline (RCS). Cross-sectional data from the National Health and Nutrition Examination Survey (NHANES, N = 4772) were then used for observational validation of the mediating pathway and analysis of gender differences. Cross-sectional NHANES data validated the mediating pathway and examined gender differences. Causal relationships of BFP with a mediator (testosterone) and outcomes (blood glucose, T1DM, T2DM) were evaluated using Mendelian randomization (MR), with inverse variance weighting (IVW) as the primary analysis. Sensitivity analyses (MR-Egger, weighted median, MR-PRESSO) assessed pleiotropy and heterogeneity.

**Results:**

Clinical data showed BFP was associated with DM (OR = 2.684, 95% CI 1.981-3.638). NHANES results indicated testosterone may mediate the BFP-DM association, accounting for 6.24% (95% CI 2.84%-10.68%) of the total effect, with significant gender differences: significant in males (14.92%, 95% CI 6.49%-25.29%) but not in females. MR analysis validated these associations: higher BFP may elevate blood glucose (OR = 1.147, 95% CI 1.097-1.200) and T2DM risk (OR = 2.456, 95% CI 2.120-2.845), while reducing testosterone (OR = 0.614, 95% CI 0.521-0.724). Mediation analysis showed testosterone partially mediated BFP’s effect on blood glucose (8.63%, 95% CI 2.19%-15.10%) and T2DM risk (3.80%, 95% CI 0.62%-6.98%), but no significant mediation was found for BFP and T1DM.

**Conclusion:**

This study speculates that testosterone partially mediates the effect of BFP on DM risk, especially in males. However, further prospective studies are needed to confirm these findings.

## Introduction

Diabetes mellitus (DM), which is broadly classified into type 1 (T1DM) and type 2 (T2DM), has emerged as a major global public health burden (1). Obesity is its primary risk factor; the 2025 World Obesity Report pointed out that 55% of disability-adjusted life years of adult T2DM attributable to risk factors are caused by high body mass index (BMI). Unlike BMI, body fat percentage (BFP) distinguishes fat from lean mass, better reflects adiposity-related metabolic dysfunction, and is a well-established indicator of T2DM risk (2). On the other hand, T1DM is an autoimmune disorder characterized by absolute insulin deficiency, but the underlying mechanisms linking it to obesity remain unclear (3). Although obesity is linked to DM, the exact biological pathways connecting adiposity to the disease, especially the distinct mechanisms in T1DM versus T2DM, are still not fully understood. Nevertheless, the mechanistic pathways linking adiposity to DM remain incompletely understood. Emerging evidence suggests that sex hormones, notably testosterone, may play a mediating role in this relationship.

As a key sex hormone, testosterone plays an important role in metabolic regulation. Obesity suppresses testosterone secretion, while hypogonadism exacerbates obesity and metabolic disorders, forming a vicious cycle (4, 5). Multiple studies have shown that decreased testosterone levels increase the risk of T2DM onset, whereas higher testosterone levels exert a protective effect (6–8). Clinical randomized controlled trials (N = 24) have confirmed that testosterone replacement therapy can improve insulin resistance and glycemic control in men with T2DM (9). Mechanistically, testosterone may be involved in biological processes such as lipid metabolism (10), inflammation (11), and insulin resistance (12). These actions suggest that it may contribute to the pathophysiology of obesity-associated DM.

Although several studies have reported these associations, the specific relationship between BFP, testosterone, and DM remains unclear. Accordingly, we hypothesized that testosterone levels mediate the BFP-DM association, with sex−specific effects. To test this hypothesis, we combined observational data from Chinese hospital records and NHANES with genetic data from European populations, enabling a multi−angle assessment of the mechanism.

## Methods

### Study design

This study integrated observational analysis with Mendelian randomization (MR) to examine whether testosterone mediates the relationship between BFP and DM. Observational data from Chinese clinical records and NHANES provided real-world association estimates. MR using European genetic instruments enabled causal inference free from unmeasured confounding and reverse causality. By comparing the mediating effects of NHANES and MR, the consistency of the results was judged, and the potential bias was evaluated. This complementary design enhances the robustness of the conclusion.

### Data sources

Clinical data were collected from the Department of Nephrology, Shengjing Hospital of China Medical University, from 1 January to 31 December 2023. A total of 4433 adult patients (age≥18 years) with complete data on all variables were enrolled using the hospital’s electronic medical record system. Details of the data filtering process are in **Supplementary Figure 1A**. The main variables included: (1) Exposure factor: BFP, calculated using the Deurenberg adult prediction formula: BFP = 1.2×BMI+0.23×Age−5.4−10.8×Sex (male=1, female=0) (13); (2) Outcome variable: DM, diagnosed in accordance with the Chinese Guidelines for the Prevention and Treatment of Diabetes (2024 Edition); (3) Covariates: age, gender, smoking, alcohol consumption, and estimated glomerular filtration rate (eGFR), calculated using the CKD-EPI formula (14).

NHANES data were collected from August 2021 to August 2023, including 4772 adult participants (age≥18 years) who completed physical measurements, laboratory tests, and questionnaires. Details of the data filtering process in **Supplementary Figure 1B**. The main study variables included: (1) BFP, calculated using the aforementioned formula; (2) Serum testosterone concentration, measured by isotope dilution liquid chromatography-tandem mass spectrometry (ID-LC-MS/MS). To minimize diurnal and postprandial variations in testosterone, the standard NHANES laboratory protocol requires participants aged 12 years and older assigned to a morning examination to fast for 9 hours before morning blood collection; (3) DM status, defined according to the American Diabetes Association diagnostic criteria as self-reported physician diagnosis, use of insulin or oral hypoglycemic agents, fasting blood glucose ≥126 mg/dL, or glycosylated hemoglobin A1c (HbA1c) ≥6.5% (15); (4) Covariates: demographic and health-related factors, including age, gender, race/ethnicity, educational level, and family income. Race/ethnicity was categorized as Mexican American, non-Hispanic Asian, non-Hispanic Black, non-Hispanic White, and other ethnic groups; educational level was divided into college and above, high school or equivalent, and below high school; family income was classified by the poverty income ratio (PIR) as low (0-1.0), medium (1.0-3.0), and high (>3.0). (5) Menopausal status was stratified based on RHQ031, RHD043, RHD280, and RHQ305: natural menopause (no regular periods for ≥12 months due to menopause, without hysterectomy or bilateral oophorectomy), surgical menopause (RHD280 = Yes or RHQ305 = Yes), and regular periods (RHQ031 = Yes) (16).

Data for MR analysis were mainly obtained from public summary statistics of large-scale genome-wide association studies (GWAS). Genetic instrumental variables for BFP were selected from the ukb-b-8909 project with a sample size of 454,633 individuals. Genetic data for testosterone levels were derived from ukb-d-30850_raw (Trait: Testosterone; Category: Metabolites), including 312,102 individuals. Genetic data related to blood glucose levels were from ebi-a-GCST90092819 with 114,870 samples. Genetic data for T1DM (4,721 cases and 403,489 controls) and T2DM (82,878 cases and 403,489 controls) were both obtained from the FinnGen database. All data included in this study were based on European populations, which effectively controlled for population stratification and provided a basis for subsequent analyses.

### Statistical analysis

#### Clinical data analysis

Continuous variables were tested for normality using the Shapiro-Wilk test (*P* < 0.05). Given that most continuous variables deviated from a normal distribution, they were presented as median with interquartile range (IQR). Categorical variables were summarized as frequencies and percentages. Differences between groups were compared using the Wilcoxon rank−sum test for continuous variables and the chi−squared test for categorical variables. Logistic regression models were used to explore the relationship between BFP and DM based on Chinese hospital clinical data. Model 1 was first constructed to consider only the direct effect of BFP on DM. Model 2 was then extended by adding gender and age as covariates. Finally, Model 3 was built by including additional covariates: age, gender, alcohol consumption, smoking, and eGFR. Subgroup analyses were performed to present the odds ratio (OR), 95% confidence interval (CI), and their significance levels. The restricted cubic spline (RCS) curve was used to analyze the relationship between changes in BFP levels and OR.

### NHANES data analysis

The descriptive statistics are consistent with the previous part. For mediation analysis, a total effect model (exposure → outcome) was first constructed, and survey-weighted generalized linear models were used to analyze the total effect of BFP on DM risk. Two mediating pathway models were then established: Model 2 (exposure → mediator) used survey-weighted linear regression to analyze the association between BFP and serum testosterone levels; Model 3 (exposure + mediator → outcome) applied survey-weighted generalized linear models to analyze the effect of testosterone on DM risk and the direct effect of BFP. Three survey-weighted regression models were fitted using the svyglm function, accounting for the complex sampling design (strata, clusters, and weights). All models were adjusted for age, race/ethnicity, educational level, and family PIR. To evaluate the impact of menopausal status, female participants were classified into natural menopause, surgical menopause, and regular periods groups. Mediation analyses were then repeated within each stratum.

The goodness of model fit was evaluated using different indicators: R^2^ and adjusted R^2^ were used for mediator models with continuous variables as outcomes (linear regression); quasi-pseudo R^2^ (calculated based on deviance) was used for DM models with binary outcomes. Multicollinearity among covariates was assessed using variance inflation factors (VIFs) based on an unweighted linear regression of testosterone on all covariates. Residuals of the mediator model were examined for normality using the Shapiro-Wilk test and for heteroscedasticity using the Breusch Pagan test. Influential observations were detected by Cook’s distance. Because the mediator model violated normality and homoscedasticity assumptions, all inference for the indirect effect relied on nonparametric bootstrapping.

Mediation analysis was performed using a nonparametric bootstrap method (1,000 resamples) with survey-weighted regression (svyglm) to account for the complex NHANES design (17). To assess the robustness of the mediation estimates, sensitivity analyses were performed using alternative bootstrap resampling numbers of 500 and 200. Effect decomposition included total effect (TE), average direct effect (ADE), and average causal mediation effect (ACME). The mediation proportion was calculated as (ACME/TE) ×100%. Percentile-based 95% confidence intervals and two-tailed p-values were derived from the bootstrap distribution. All analyses were stratified by sex to explore gender specificity.

### MR analysis

The screening threshold for instrumental variables in this study was *P* < 5×10^−8^, and linkage disequilibrium analysis (window size: 10,000 kb, *r^2^* < 0.001) was performed to ensure the independence of genetic variants (the screening threshold for T2DM was *P* < 5×10^−9^). All included single-nucleotide polymorphisms (SNPs) were evaluated using the F statistic. Only instrumental variables with an F statistic greater than 10 were retained (**Supplementary Table 1**), which is the conventional threshold for excluding weak instruments. The exposure and outcome datasets were merged and harmonized using the TwoSampleMR package. The primary analysis was based on the IVW method (fixed and random effects). To address potential horizontal pleiotropy, we additionally applied weighted median (consistent if ≥50% valid instruments), weighted mode (unbiased when the largest cluster of estimates is valid), and MR-Egger (detects directional pleiotropy). Consistency across methods was interpreted as supporting causal robustness (18).

Comprehensive sensitivity analyses were implemented: the MR-PRESSO method (10,000 iterations, significance threshold: 0.1) was used to identify and exclude outliers. This threshold was selected to maintain adequate power for detecting horizontal pleiotropy while identifying and removing pleiotropic outliers, thereby avoiding false−negative outliers. Cochran’s Q test was employed to assess heterogeneity among instrumental variables; the intercept term of the MR-Egger regression was used to test horizontal pleiotropy; the leave-one-out method was applied to determine whether the results were overly driven by a single strong instrumental variable.

The mediating effect was evaluated using a two-step MR framework (19). The first step established an independent causal chain from BFP to testosterone (exposure-mediator path β_1_). The second step analyzed the mediator-outcome path (β_2_) from testosterone to DM, where SNPs that were used in the first step (SPNs associated with BFP) were excluded to prevent overlap. Finally, the causal association from BFP to DM was analyzed (total effect path β). The point estimate of the mediating effect was obtained by the product of path coefficients (β_1_β_2_), and its standard error was calculated using the Sobel formula: ${\text{SE }}_{Sobel}=(\sqrt{{\text{β}}_{1}{)}^{2}•({\text{SE}}_{2}{)}^{2}+({\text{β}}_{2}{)}^{2}•({\text{SE}}_{1}{)}^{2}}$. The direct effect was estimated by the difference between the total effect and the mediating effect (β_3_=β−β_1_β_2_), and its standard error was derived from the sum of the variances of the total effect and the mediating effect. The mediation proportion was calculated as the ratio of the mediating effect to the total effect (β_1_β_2_/β) and presented as a percentage. All statistical analyses were completed using R software (version 4.5.1).

## Results

### Results of Chinese clinical data analysis

A total of 4433 participants were enrolled in this study, including 975 DM patients and 3458 controls. Baseline characteristics showed that the participants were aged 18–94 years, with a median age of 56 (43, 65) years; 58.3% were male, and 41.7% were female. BFP was significantly higher in the DM group than in the control group (*P* < 0.001), and there were also differences in age, smoking, alcohol, and eGFR between the two groups (**Table 1**).

**Table 1:** Baseline characteristics of the study population in Chinese clinical data.

Variables	Overall	Non-Diabetic	Diabetes	*P*
N	4433	3458	975	
BFP (median [IQR])	29.589 [25.263, 35.536]	29.328 [24.910, 34.928]	30.921 [26.200, 37.602]	<0.001
Age (median [IQR])	56.000[43.000, 65.000]	54.000 [41.000, 64.000]	60.000 [51.000, 67.000]	<0.001
eGFR (median [IQR])	64.649 [16.212, 97.704]	68.434 [19.691, 99.753]	49.351 [11.934, 89.264]	<0.001
Gender (%)				
Female	1847 (41.7)	1461 (42.2)	386 (39.6)	0.147
Male	2586 (58.3)	1997 (57.8)	589 (60.4)	
Smoking (%)				
No	2843 (64.1)	2245 (64.9)	598 (61.3)	0.043
Yes	1590 (35.9)	1213 (35.1)	377 (38.7)	
Alcohol (%)				
No	3336 (75.3)	2628 (76.0)	708 (72.6)	0.034
Yes	1097 (24.7)	830 (24.0)	267 (27.4)	

BFP was then divided into four groups by quartiles: Q1 (<25.263%), Q2 (25.263% to<29.589%), Q3 (29.589% to<35.536%), and Q4 (≥35.536%). In different logistic regression models (**Table 2**), high BFP (Q2, Q3, Q4) was associated with an increased odds of DM compared with Q1. Multivariate logistic regression analysis showed that after adjusting for all covariates, higher BFP (Q4) was still significantly associated with an increased odds of DM (OR = 2.684, 95% CI 1.981-3.638, *P* < 0.001) (**Supplementary Table 2**).

**Table 2:** Logistic regression analysis of the association between BFP quartiles and DM risk in Chinese clinical data.

BFP	Diabetes		Model 1				Model 2				Model 3			
	Yes	No	OR	95%CI low	95%CI up	*P*	OR	95%CI low	95%CI up	*P*	OR	95%CI low	95%CI up	*P*
Q1: < 25.263 %	186	923	Reference											
Q2: 25.263 to < 29.589 %	246	862	1.416	1.146	1.750	0.001	1.244	1.000	1.548	0.050	1.280	1.027	1.594	0.028
Q3: 29.589 to < 35.536 %	227	882	1.277	1.030	1.583	0.026	1.399	1.100	1.778	0.006	1.438	1.130	1.830	0.003
Q4: ≥ 35.536 %	316	791	1.982	1.616	2.432	<0.001	2.571	1.898	3.481	<0.001	2.684	1.981	3.638	<0.001

**Figure 1:**
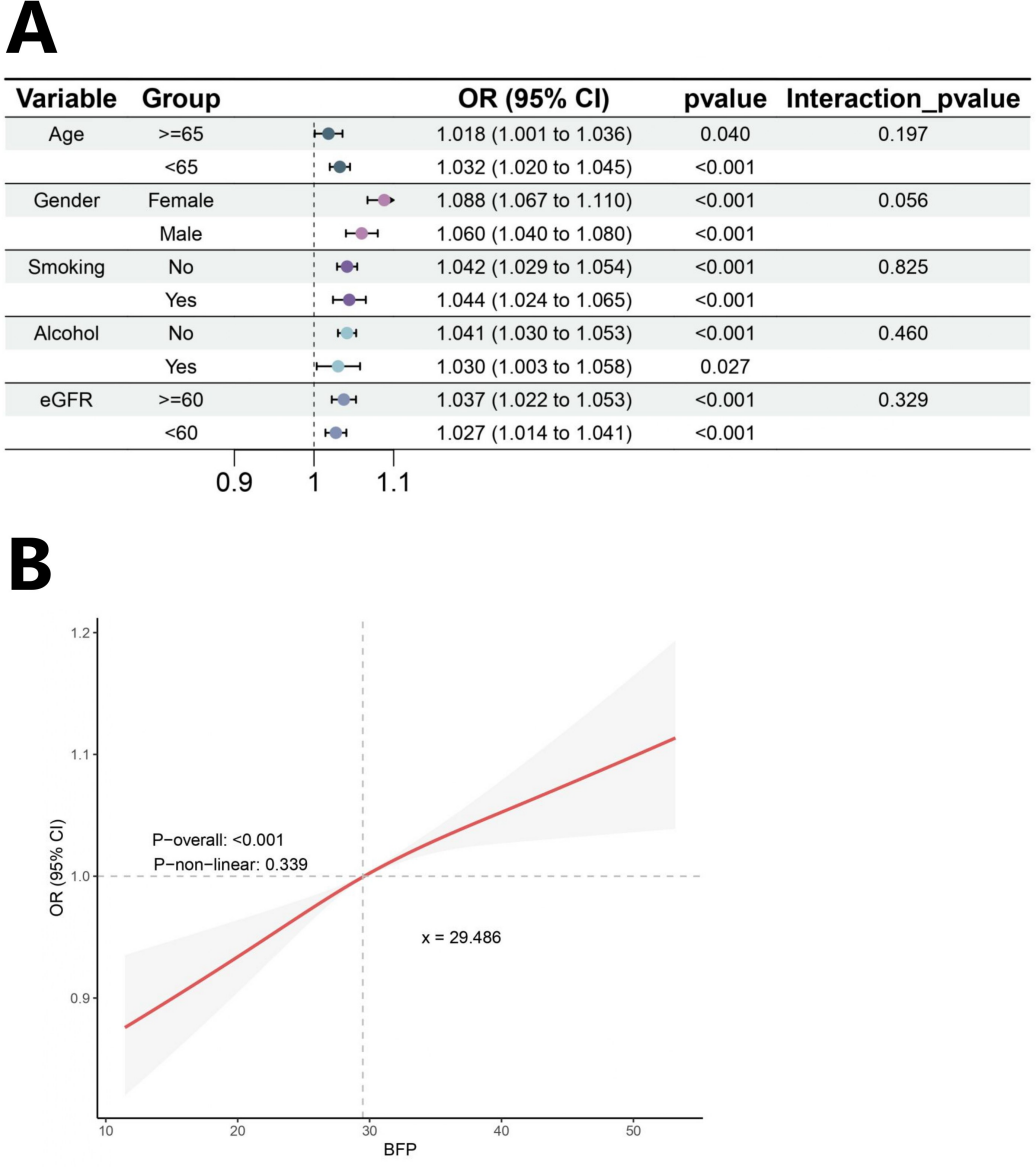
Association between body fat percentage and diabetes mellitus risk. **(A)** Forest plot of subgroup analyses. **(B)** RCS curve depicting the dose-response relationship.

Subgroup analysis suggested that BFP was positively correlated with the risk of DM in all stratifications of age, gender, smoking, alcohol consumption, and eGFR, with no significant interaction observed (*P*>0.05) (**Figure 1A**). RCS analysis further confirmed the association between BFP and DM (*P* < 0.001). However, the nonlinearity test was not significant (*P* = 0.339), indicating that the relationship is predominantly linear across the observed BFP range. The OR value was 1 when BFP was 29.486%, and the odds of DM increased with the elevation of BFP (**Figure 1B**). This point approximates the median value, which provides a reference for estimating the relative advantage of the BFP spectrum.

### Results of NHANES data analysis

#### Sample characteristics of the study population

A total of 4772 participants were included in the study (**Table 3**), among whom 2622 (54.9%) were female, and 2150 (45.1%) were male. The participants were aged 20 to 80 years, with a median age of 57 (39, 68) years. In terms of racial distribution, non-Hispanic Whites were the predominant group (61.2%). Regarding educational level, most participants had received higher education (68.3% with college and above degrees). According to poverty classification, the proportions of participants with high, medium, and low PIR were 48.6%, 36.8%, and 14.7%, respectively. All the above demographic characteristics showed significant differences between the DM group and the control group.

**Table 3:** Baseline characteristics of the study population in NHANES data.

Variable	ALL				Female				Male			
	Overall	Non-Diabetic	Diabetes	*P*	Overall	Non-Diabetic	Diabetes	*P*	Overall	Non-Diabetic	Diabetes	*P*
N	4772	3878	894		2622	2164	458		2150	1714	436	
BFP (median [IQR])	36.770 [30.110, 44.810]	35.665 [28.980, 43.333]	42.790 [35.323, 51.278]	<0.001	42.400 [36.052, 49.412]	40.770 [34.820, 47.275]	50.065 [44.000, 55.908]	<0.001	31.030 [25.722, 36.415]	29.730 [24.497, 34.745]	35.950 [31.262, 40.642]	<0.001
Testosterone (median [IQR])	1.550 [0.672, 14.200]	1.520 [0.691, 14.600]	1.730 [0.584, 12.075]	<0.001	0.727 [0.480, 1.080]	0.753 [0.500, 1.110]	0.600 [0.399, 0.898]	<0.001	14.900 [11.000, 19.200]	15.500 [11.600, 20.075]	12.200 [8.980, 16.300]	<0.001
Age (median [IQR])	57.000 [39.000, 68.000]	54.000 [37.000, 66.000]	64.000 [56.000, 71.750]	<0.001	56.000 [39.000, 67.000]	53.000 [37.000, 66.000]	63.000 [55.000, 71.000]	<0.001	58.000 [40.000, 68.000]	55.000 [37.000, 67.000]	65.000 [57.000, 72.000]	<0.001
Gender (%)												
Female	2622 (54.9)	2164 (55.8)	458 (51.2)	0.015								
Male	2150 (45.1)	1714 (44.2)	436 (48.8)								
Race (%)												
Mexican American	312 (6.5)	234 (6.0)	78 (8.7)	<0.001	160 (6.1)	116 (5.4)	44 (9.6)	<0.001	152 (7.1)	118 (6.9)	34 (7.8)	0.064
Non-Hispanic Asian	252 (5.3)	215 (5.5)	37 (4.1)		140 (5.3)	122 (5.6)	18 (3.9)		112 (5.2)	93 (5.4)	19 (4.4)	
Non-Hispanic Black	518 (10.9)	390 (10.1)	128 (14.3)		289 (11.0)	219 (10.1)	70 (15.3)		229 (10.7)	171 (10.0)	58 (13.3)	
Non-Hispanic White	2922 (61.2)	2439 (62.9)	483 (54.0)		1613 (61.5)	1374 (63.5)	239 (52.2)		1309 (60.9)	1065 (62.1)	244 (56.0)	
Other Race	768 (16.1)	600 (15.5)	168 (18.8)		420 (16.0)	333 (15.4)	87 (19.0)		348 (16.2)	267 (15.6)	81 (18.6)	
Education (%)												
College or above	3259 (68.3)	2762 (71.2)	497 (55.6)	<0.001	1853 (70.7)	1599 (73.9)	254 (55.5)	<0.001	1406 (65.4)	1163 (67.9)	243 (55.7)	<0.001
High school or equivalent	965 (20.2)	762 (19.6)	203 (22.7)		492 (18.8)	389 (18.0)	103 (22.5)		473 (22.0)	373 (21.8)	100 (22.9)	
Less than high school	548 (11.5)	354 (9.1)	194 (21.7)		277 (10.6)	176 (8.1)	101 (22.1)		271 (12.6)	178 (10.4)	93 (21.3)	
Poverty (%)												
High	2317 (48.6)	1976 (51.0)	341 (38.1)	<0.001	1193 (45.5)	1048 (48.4)	145 (31.7)	<0.001	1124 (52.3)	928 (54.1)	196 (45.0)	0.003
Low	701 (14.7)	541 (14.0)	160 (17.9)		419 (16.0)	326 (15.1)	93 (20.3)		282 (13.1)	215 (12.5)	67 (15.4)	
Medium	1754 (36.8)	1361 (35.1)	393 (44.0)		1010 (38.5)	790 (36.5)	220 (48.0)		744 (34.6)	571 (33.3)	173 (39.7)	

For the exposure variable, the median BFP of all participants was 36.770 [30.110, 44.810], and BFP was significantly higher in the DM group (*P* < 0.001). BFP exhibited gender differences: the median value was 31.030 [25.722, 36.415] in males and 42.400 [36.052, 49.412] in females. The median level of the mediator (testosterone) was 1.550 [0.672, 14.200] nmol/L. Testosterone levels also showed obvious gender differences, with the median value in males (14.900nmol/L) much higher than that in females (0.727nmol/L). In the overall population, the median testosterone level in the DM group (1.730nmol/L) was slightly higher than in the control group (1.520nmol/L), whereas after sex stratification, both male and female subgroups showed lower testosterone levels in the DM group (**Table 3**). This discrepancy arises from confounding by gender—females have substantially lower testosterone levels than males, and the proportion of females differed between the DM and control groups. Therefore, subsequent analyses were conducted for the full sample as well as for male and female subgroups separately.

#### Results of NHANES mediation analysis

**Figure 2:**
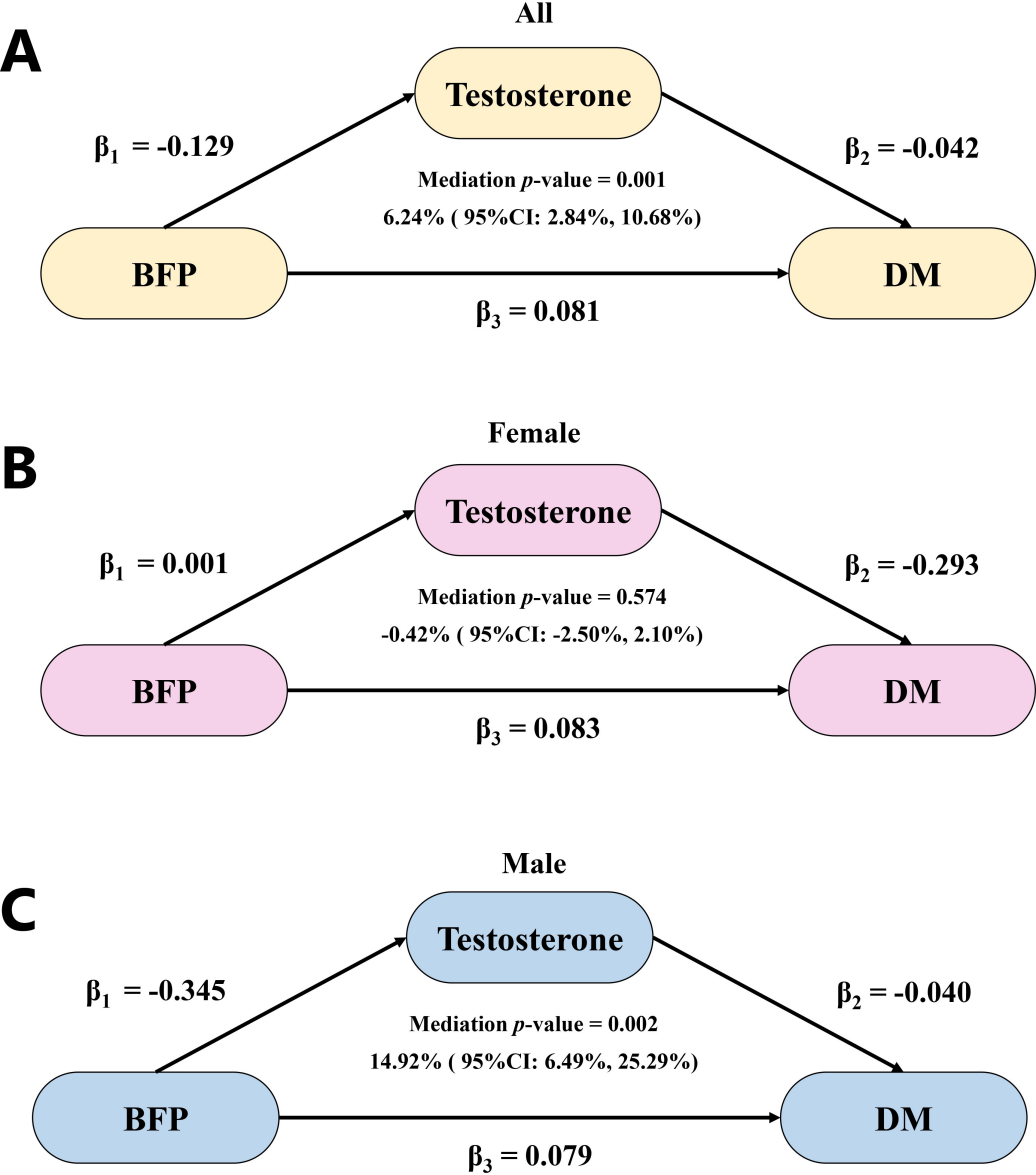
Mediation analysis diagrams based on NHANES data. **(A)** Path diagram for the overall population. **(B)** Path diagram for the female subgroup. **(C)** Path diagram for the male subgroup.

Model diagnostics showed no substantial multicollinearity among covariates (all VIF< 2) and no influential observations based on Cook’s distance (maximum< 1). Although the residuals of the linear mediation model deviated slightly from normality and exhibited heteroscedasticity, the nonparametric bootstrap method we used does not rely on these assumptions, thereby ensuring the robustness of the inference (**Supplementary Table 3**). The mediating role of testosterone in the association between BFP and DM risk was explored in the total population (**Figure 2A**; **Table 4**). After adjusting for covariates (age, gender, race, educational level, poverty level), BFP showed a significant negative association with testosterone levels (β=−0.129, *P* < 0.001), and testosterone was also significantly negatively associated with DM (β=−0.042, *P* = 0.047). The ACME was 0.005 (95% CI 0.002-0.009, *P* < 0.001), accounting for an estimated 6.24% of the total effect (95% CI 2.84%-10.68%, *P* = 0.001) (**Table 5**). Given that the *P* value of the effect of testosterone on DM was close to the significance threshold, the estimation of the mediating effect should be interpreted with caution, and its stability needs further verification in larger samples. In addition, the adjusted R^2^ of the model for predicting testosterone levels in this study was 0.620, with a β value of gender of 13.480 (*P* < 0.001), suggesting that gender is a strong determinant of testosterone levels. Therefore, further gender-stratified analyses were performed.

**Table 4:** Mediation analysis of testosterone in the association between BFP and DM in NHANES.

Group	Variables	Model 1 (Diabetes)				Model 2 (Testosterone)				Model 3 (Diabetes)			
		β	SE	*t*	*P*	β	SE	*t*	*P*	β	SE	*t*	*P*
ALL (N=4772)	Intercept	-7.528	0.286	-26.327	<0.001	7.092	0.865	8.195	0.001	-7.284	0.286	-25.500	<0.001
	X (BPF)	0.085	0.005	18.291	<0.001	-0.129	0.008	-15.143	<0.001	0.081	0.004	19.166	<0.001
	M (Testosterone)									-0.042	0.013	-3.272	0.047
	R^2^	0.084				0.621				0.089			
	Adjusted R^2^	\				0.620				\			
Female (N=2622)	Intercept	-7.072	0.539	-13.117	<0.001	1.752	0.368	4.761	0.005	-6.788	0.549	-12.354	<0.001
	X (BPF)	0.082	0.006	13.035	<0.001	0.001	0.003	0.369	0.727	0.083	0.006	13.961	<0.001
	M (Testosterone)									-0.293	0.165	-1.778	0.150
	R^2^	0.091				0.035				0.095			
	Adjusted R^2^	\				0.031				\			
Male (N=2150)	Intercept	-6.680	0.476	-14.040	<0.001	25.996	1.784	14.571	<0.001	-5.795	0.553	-10.485	<0.001
	X (BPF)	0.089	0.009	9.843	<0.001	-0.345	0.025	-13.624	<0.001	0.079	0.008	10.455	<0.001
	M (Testosterone)									-0.040	0.013	-3.211	0.033
	R^2^	0.073				0.112				0.081			
	Adjusted R^2^	\				0.107				\			

**Table 5:** Effects and mediation proportions from the mediation analysis in NHANES.

Type	All (N=4772)				Female (N=2622)				Male (N=2150)			
	Estimate	95% CI-low	95% CI-up	*P*	Estimate	95% CI-low	95% CI-up	*P*	Estimate	95% CI-low	95% CI-up	*P*
ACME (average)	0.005	0.002	0.009	<0.001	-0.0003	-0.002	0.002	0.569	0.014	0.006	0.024	0.007
ADE (average)	0.081	0.070	0.093	<0.001	0.083	0.069	0.100	<0.001	0.079	0.063	0.099	<0.001
Total Effect	0.087	0.076	0.099	<0.001	0.083	0.069	0.100	<0.001	0.093	0.076	0.113	<0.001
Mediated (%)	6.24%	2.84%	10.68%	0.001	-0.42%	-2.50%	2.10%	0.574	14.92%	6.49%	25.29%	0.002

In the female group (**Figure 2B**; **Table 4**), the effect of BFP on testosterone levels was small and non-significant (β=0.001, *P* = 0.727). In Model 3, the effect of testosterone on DM was also non-significant (β=−0.293, *P* = 0.150), while BFP was positively associated with DM (β=0.083, *P* < 0.001). The ACME was -0.0003 (95% CI -0.002-0.002, *P* = 0.569), with a mediation proportion of −0.42% (*P* = 0.574). After stratifying females into natural menopause (ACME = −0.001, P = 0.380), surgical menopause (ACME = 0.002, P = 0.410), and regular periods (ACME = 0.0003, *P* = 0.609), the mediating effect of testosterone remained non−significant in three subgroups (**Supplementary Table 7**). These findings suggest that the mediating role of testosterone in the association between BFP and DM was not significant in females (**Table 5**). Given the low circulating testosterone levels in females and their susceptibility to interference from various physiological factors, further research is required to explore testosterone-related effects in females.

In the male group (**Figure 2C**; **Table 4**), BFP was positively correlated with DM (β=0.089, *P* < 0.001). Unlike in females, BFP had a significant negative effect on testosterone levels (β=−0.345, *P* < 0.001). Meanwhile, testosterone levels were negatively associated with DM (β=−0.040, *P* = 0.033), and BFP was still directly positively correlated with DM (β=0.079, *P* < 0.001). Mediation analysis suggested that the mediating effect of testosterone between BFP and DM was significant in males. The ACME was 0.014 (95% CI 0.006-0.024, *P* = 0.007), accounting for 14.92% of the total effect (95% CI 6.49%-25.29%, *P* = 0.002). Sensitivity analyses using bootstrap resampling numbers (200 and 500) consistently demonstrated that the mediating effect of testosterone remained statistically significant in both the overall population and male group (**Supplementary Table 8**). This result suggested that BFP may influence the risk of DM partly through lowering testosterone levels in males (**Table 5**). Detailed model parameters are shown in **Supplementary Tables 4**–**6**. The biological mechanisms underlying the mediating effects in male merit further discussion.

### Results of MR analysis

#### Causal relationship between BFP and DM

**Figure 3:**
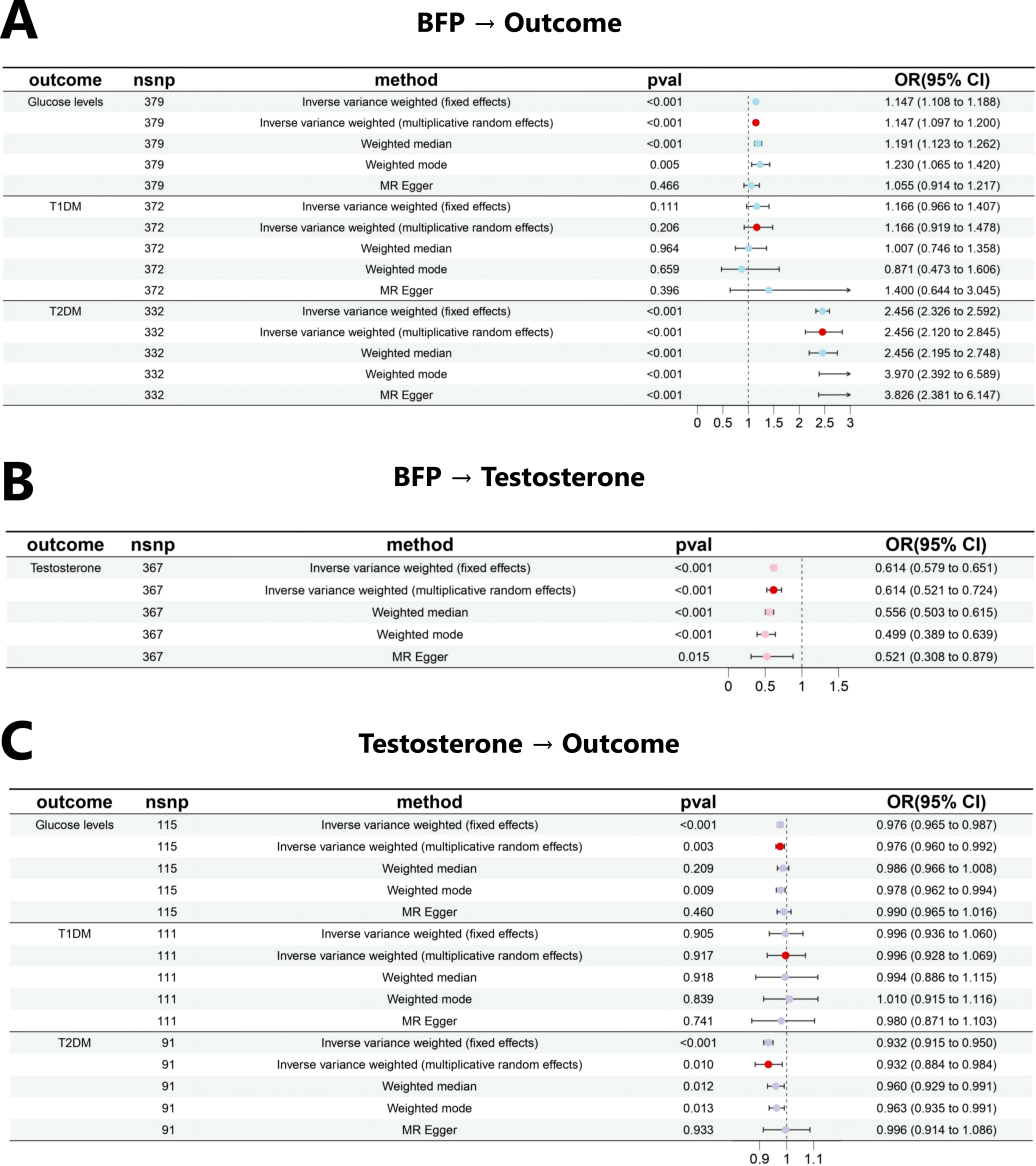
Forest plots of Mendelian randomization analyses. **(A)** Mendelian randomization result of BFP on DM risk. **(B)** Mendelian randomization result of BFP on testosterone levels. **(C)** Mendelian randomization result of testosterone levels on DM risk.

MR analysis was used to explore the causal relationship between BFP and DM (**Figure 3A**). Since heterogeneity was still detected after excluding abnormal SNPs by the MR-PRESSO method (**Table 6**), the random-effects model was selected for causal inference. Results of the IVW random-effects model showed that higher BFP may be a risk factor for elevated blood glucose levels (OR = 1.147, 95% CI 1.097-1.200) and increased risk of T2DM (OR = 2.456, 95% CI 2.120-2.845), but no significant causal association was found with T1DM (**Figure 3A**; **Supplementary Table 9**). Further sensitivity analyses supported the robustness of the results (**Table 6**; **Supplementary Figure 2**). The intercept term test of MR-Egger regression indicated no significant genetic horizontal pleiotropy in the analyses of blood glucose levels and T2DM (*P*>0.05). The leave-one-out analysis also confirmed that the causal estimates were not overly driven by a single strong instrumental variable (**Supplementary Figure 3**–**5**).

**Table 6:** Sensitivity analyses of the Mendelian randomization study.

Exposure	Outcome	MR-PRESSO			Pleiotropy			Heterogeneity		
		RSS obs	Outliers	*P*	Intercept	SE	*P*	Method	Q	*P*
Body fat percentage	Glucose levels	616.761	3	<0.001	0.001	0.001	0.227	MR Egger	608.393	<0.001
								IVW	610.752	<0.001
	T1DM	593.896	2	<0.001	-0.003	0.005	0.627	MR Egger	589.013	<0.001
								IVW	589.389	<0.001
	T2DM	3080.127	42	<0.001	-0.006	0.003	0.055	MR Egger	2413.651	<0.001
								IVW	2440.798	<0.001
	Testosterone	2905.983	16	<0.001	0.002	0.004	0.515	MR Egger	2858.554	<0.001
								IVW	2861.877	<0.001
Testosterone	Glucose levels	283.517	3	<0.001	-0.002	0.001	0.168	MR Egger	246.585	<0.001
								IVW	250.778	<0.001
	T1DM	317.263	4	<0.001	0.002	0.005	0.737	MR Egger	141.987	0.018
								IVW	142.135	0.021
	T2DM	1250.844	24	<0.001	-0.007	0.004	0.060	MR Egger	741.790	<0.001
								IVW	772.006	<0.001
Glucose levels	Body fat percentage	190.874	7	<0.001	0.001	0.002	0.661	MR Egger	35.184	0.004
								IVW	35.623	0.005
T1DM		119.670	3	<0.001	-0.001	0.002	0.656	MR Egger	106.585	<0.001
								IVW	107.672	<0.001
T2DM		3206.467	49	<0.001	0.003	0.002	0.072	MR Egger	2272.816	<0.001
								IVW	2333.308	<0.001

To assess potential reverse causality, reverse MR analysis was performed (**Table 7**; **Supplementary Figures 2, 6–8**). Due to heterogeneity (**Table 6**), the IVW random-effects model was mainly used for analysis. Results showed no significant causal associations between genetically predicted blood glucose levels, T1DM, and BFP. However, in the analysis of the effect of T2DM on BFP, the IVW random-effects model showed a statistically significant association when the instrumental variable screening threshold was *P* < 5×10^−8^, but this result was affected by genetic horizontal pleiotropy, and different MR analysis methods yielded inconsistent effect directions, indicating limited reliability. To explore the robustness of this association, the screening strictness of instrumental variables was gradually increased (5×10^−8^, 1×10^−8^, 5×10^−9^, 1×10^−9^, 5×10^−10^, and 1×10^−10^). Sensitivity analysis showed that when the screening threshold was strictly set to *P* < 5×10^−9^, the IVW model no longer showed a significant association, and the horizontal pleiotropy test turned negative (**Supplementary Table 10**). Although a loose instrumental variable threshold suggested an association between T2DM and BFP, this result could not be replicated in pleiotropy tests and more rigorous analyses. We finally selected *P* < 5×10^−9^ as the instrumental variable screening condition for T2DM. The existing results do not support the reverse causality hypothesis that T2DM is a risk factor for BFP, and the causal relationship between them needs further evidence for verification.

**Table 7:** Detailed results of the reverse Mendelian randomization analysis.

Outcome	Exposure	Method	nSNP	β	SE	*P*	OR	OR 95%CI low	OR 95%CI up
Body fat percentage	Glucose levels	IVW (fixed effects)	18	-0.023	0.012	0.053	0.977	0.954	1.000
		IVW (random effects)	18	-0.023	0.017	0.181	0.977	0.945	1.011
		Weighted median	18	-0.020	0.019	0.276	0.980	0.945	1.016
		Weighted mode	18	-0.017	0.026	0.514	0.983	0.935	1.034
		MR Egger	18	-0.046	0.055	0.410	0.955	0.858	1.063
	T1MD	IVW (fixed effects)	22	-0.004	0.001	<0.001	0.996	0.993	0.998
		IVW (random effects)	22	-0.004	0.002	0.075	0.996	0.991	1.000
		Weighted median	22	-0.006	0.002	<0.001	0.994	0.991	0.997
		Weighted mode	22	-0.003	0.002	0.048	0.997	0.994	1.000
		MR Egger	22	-0.003	0.004	0.472	0.997	0.989	1.005
	T2DM	IVW (fixed effects)	126	0.019	0.003	<0.001	1.019	1.014	1.024
		IVW (random effects)	126	0.019	0.011	0.095	1.019	0.997	1.042
		Weighted median	126	-0.014	0.006	0.030	0.987	0.974	0.999
		Weighted mode	126	-0.028	0.006	<0.001	0.972	0.961	0.984
		MR Egger	126	-0.018	0.023	0.440	0.982	0.939	1.028

#### Causal relationship between BFP and testosterone

Applying the MR-PRESSO method (10,000 iterations, outlier significance threshold *P* < 0.1), sixteen abnormal SNPs were detected and excluded. Due to heterogeneity (*P* < 0.001) (**Table 6**), results were mainly inferred based on the IVW random-effects model. Analysis showed that higher BFP may have a causal association with lower circulating testosterone levels (OR = 0.614, 95% CI 0.521-0.724) (**Figure 3B**). The intercept term test of MR-Egger regression found no significant evidence of genetic horizontal pleiotropy (*P* = 0.515), and the leave-one-out sensitivity analysis further confirmed the robustness of the results (**Supplementary Figure 9**).

#### Causal relationship between testosterone and DM

Results of the IVW random-effects model suggested that higher testosterone levels may be a protective factor for reduced blood glucose (OR = 0.976, 95% CI 0.960-0.992) and decreased risk of T2DM (OR = 0.932, 95% CI 0.884-0.984), but no significant causal association was found with T1DM risk (**Figure 3C**). The intercept term test of MR-Egger regression for blood glucose levels and T2DM found no significant horizontal pleiotropy (*P*>0.05) (**Table 6**), and the leave-one-out sensitivity analysis further indicated the robustness of the results (**Supplementary Figures 10–12**).

#### Results of mediation effect analysis

**Figure 4:**
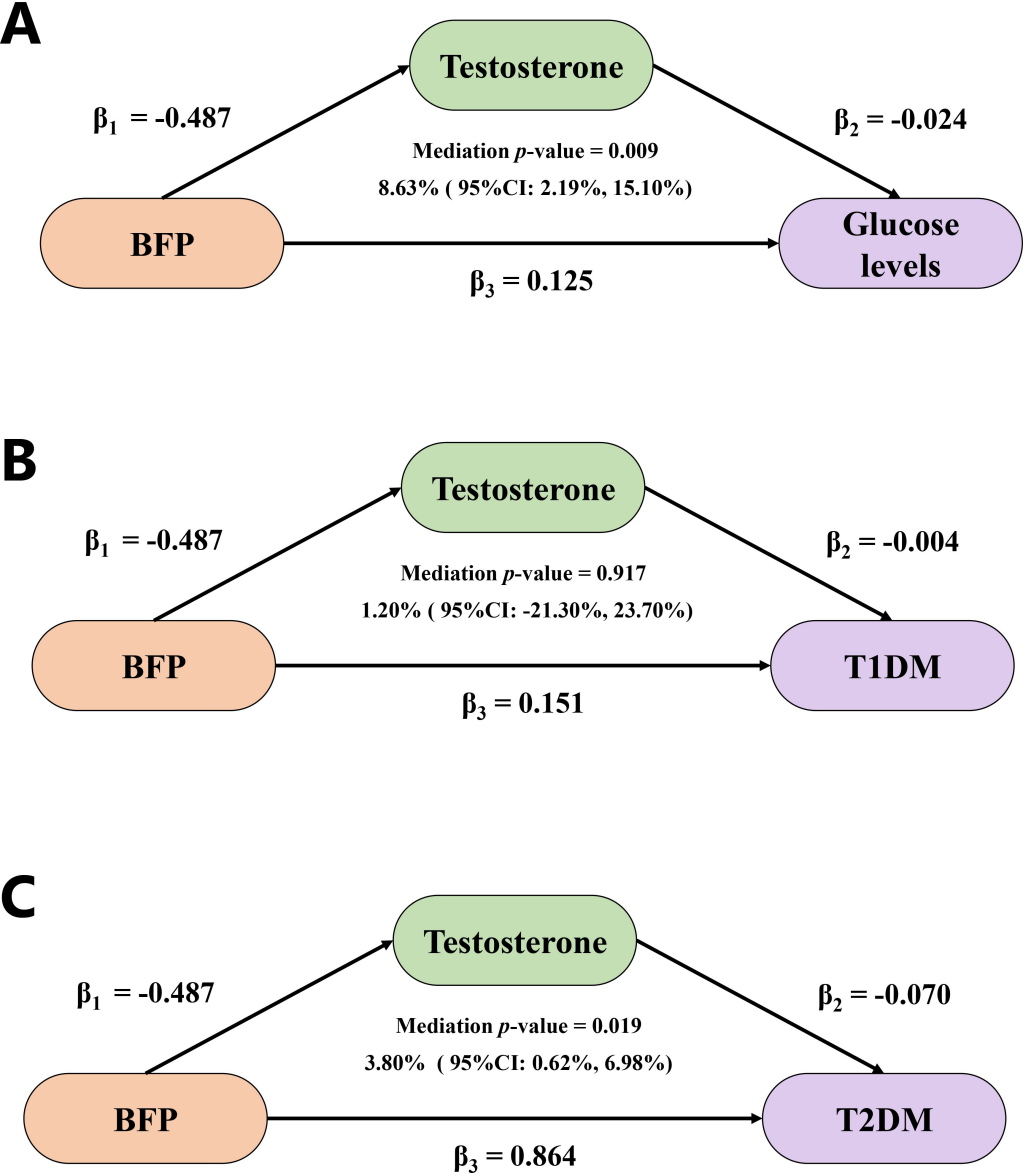
Path diagrams of the mediation analysis from Mendelian randomization. **(A)** Mediation pathway for glucose levels. **(B)** Mediation pathway for T1DM. **(C)** Mediation pathway for T2DM.

Mediation effect analysis was performed to explore whether testosterone plays a mediating role between BFP and DM. As shown in **Figure 4**, BFP harmed testosterone levels β_1_=−0.487). In the mediating pathway of blood glucose levels (**Figure 4A**), testosterone exhibited a significant mediating effect (*P* = 0.009), with a mediation proportion of 8.63% (95% CI 2.19%-15.10%). In T1DM (**Figure 4B**), the mediating effect was not statistically significant (*P* = 0.917), with an effect proportion of 1.20% (95% CI -21.30%-23.70%). In T2DM (**Figure 4C**), the mediating effect was significant (*P* = 0.019), with a mediation proportion of 3.80% (95% CI 0.62%-6.98%), suggesting that testosterone also plays a partial mediating role in the association between BFP and T2DM, although its contribution is relatively low.

## Discussion

This study explored the association between BFP and DM by integrating Chinese clinical data, US NHANES data, and GWAS data from European populations, and systematically evaluated the mediating role of testosterone in the relationship between BFP and T2DM risk, with this effect showing gender differences. The results of this study suggest that higher BFP may indirectly increase the risk of DM by reducing circulating testosterone levels, especially in males.

### Potential biological mechanisms of the mediating effect of testosterone

From a biological mechanism perspective, BFP may regulate testosterone levels through multiple pathways (20). Adipose tissue dysfunction caused by obesity, including leptin resistance, increased aromatase activity, and release of inflammatory factors, can impair the function of the hypothalamic-pituitary-testicular axis through various means, thereby leading to decreased testosterone levels. At the same time, hypogonadism may further promote fat accumulation, thus forming a vicious cycle (21, 22). According to previous studies, leptin is mainly secreted by adipocytes and acts on the hypothalamus to regulate appetite, energy expenditure, and reproductive function (23). It has also been reported that leptin can act on the testis and negatively regulate testosterone synthesis (24). Adipose tissue is also rich in aromatase, an enzyme that converts testosterone to estrone (25). Studies have shown that in obese males, increased aromatase activity in subcutaneous adipose tissue reduces testosterone levels (10). In addition, accumulating evidence indicates that inflammatory status also has an inhibitory effect on testosterone synthesis. In adipose tissue, adipocytes and preadipocytes can synthesize and release a variety of cytokines, such as Tumor Necrosis Factor-alpha (TNF-α), Interleukin-1 beta (IL-1β), and Interleukin-6 (IL-6) (26). In addition, testosterone deficiency is associated with elevated levels of certain proinflammatory cytokines (like IL-1β, IL-6, and TNF-α) (11). Previous research has found that chronic inflammation may inhibit the function of Leydig cells through these cytokines, thereby downregulating the synthesis of male steroid hormones (27). Other studies have suggested that low testosterone may promote insulin resistance, and insulin resistance (especially accompanied by obesity) may also inhibit testosterone synthesis (28). For instance, it has been shown that testosterone can enhance the efficiency of the insulin signaling pathway and reduce insulin resistance in peripheral tissues (12). Studies have demonstrated that testosterone improves the body’s insulin sensitivity overall by increasing muscle mass (the main insulin-sensitive tissue) and reducing visceral fat (the main source of inflammation and insulin resistance) (29). Additionally, testosterone has been reported to promote protein synthesis and maintain or increase muscle mass (30, 31). It is well established that muscle is the main site for glucose consumption, and increased muscle mass helps stabilize blood glucose (32). Taken together, these literature-based mechanisms support that the inferences of this study are consistent with most observational studies and experimental mechanism studies, further indicating that testosterone is a mediating factor linking abnormal body fat and glucose metabolic disorders. Although these mechanisms were not directly evaluated in the present study, they provide a plausible biological basis for the observed associations. The above mechanistic interpretation is speculative and requires validation in future research.

### Discussion on gender differences

This study found that the mediating effect of testosterone in the association between BFP and DM may exhibit sexual dimorphism. In males, low testosterone levels appear to serve as an intermediate link in the pathway from high BFP to increased DM risk, whereas no statistically significant mediating effect of testosterone was observed in females. Although these findings are based on observational studies and MR analysis, the underlying mechanisms remain to be elucidated. Accumulating evidence indicates that the effects of testosterone and its relationship with adiposity are sexually dimorphic. In males, higher testosterone levels are associated with a lower risk of T2DM, suggesting a protective role, whereas in females, higher testosterone levels are linked to increased metabolic risk (33, 34). Additionally, adiposity indicators are negatively correlated with testosterone levels in males (35). The relationship in females is more complex: bioavailable testosterone is positively correlated with total BFP, while total testosterone is negatively correlated (36).

The mechanisms underlying sex differences involve sex hormone−binding globulin (SHBG), fat distribution, insulin resistance, and pancreatic β−cell function, etc. SHBG levels are influenced by both obesity and sex (37, 38). SHBG is part of the testosterone regulatory system; adjusting for it may lead to overadjustment. Therefore, future studies directly measuring free testosterone are needed to validate our findings. Furthermore, testosterone stimulates insulin secretion and antagonizes β−cell apoptosis, with a sex−dimorphic effect: physiological testosterone in males enhances GLP−1 signaling to exert anti−diabetic effects, whereas excessively high testosterone in females may induce oxidative damage and β−cell dysfunction (39–41). In females, a sharp postmenopausal decline in estrogen leads to central fat distribution and abnormal lipolysis, promoting insulin resistance (42, 43). In our study, after stratifying by menopausal status, the mediating effect of testosterone was not statistically significant in any female subgroup. It indicates that, regardless of menopausal status, testosterone is not a major mediator of the BFP−diabetes association in females. The relatively small sample sizes do not completely exclude a weak modifying role of menopause, and larger prospective cohorts are needed to validate these findings.

### Divergent mediating roles in T1DM and T2DM

Our MR analysis identified higher BFP as a risk factor for elevated blood glucose (OR = 1.147) and T2DM (OR = 2.456), whereas higher testosterone levels were protective against both (glucose: OR = 0.976; T2DM: OR = 0.932). Our MR sensitivity analyses supported the robustness of our causal estimates. The leave−one−out analysis demonstrated that no single SNP disproportionately influenced the result, as the causal estimate remained stable after sequentially removing each SNP. Furthermore, the MR−Egger intercept showed no evidence of significant horizontal pleiotropy, and the direction of the causal estimates was consistent across different MR methods. Mediation analysis revealed that testosterone partially mediated the BFP-diabetes association, with a mediation proportion for blood glucose (8.63%) than for T2DM (3.80%). However, it can be observed from the β that BFP and testosterone may have a stronger effect on T2DM than glucose. This indicates that their effects on glucose homeostasis accumulate over time, making T2DM diagnosis a better indicator of long‑term effects than a single glucose measurement. The larger total effect of BFP on T2DM also explains the lower mediation proportion for T2DM, as a larger denominator reduces the relative contribution of the indirect effect. In contrast, no significant mediation was observed for T1DM (proportion mediated 1.20%). T1DM is an autoimmune disorder characterized by absolute insulin deficiency (44), whereas T2DM involves insulin resistance and metabolic dysfunction, and is more associated with obesity and testosterone (45–47). The null finding for T1DM, while consistent with this pathophysiological distinction, requires confirmation in future studies.

Novel therapeutic strategies for diabetes and its comorbidities also merit attention. For instance, remote monitoring has demonstrated significant potential in improving glycemic control and lipid profiles in diabetic patients (48). Accumulating evidence links testosterone status to chronic diseases (49, 50), including cardiovascular disorders and kidney diseases, etc. Our findings are supported by recent advances in diabetes therapeutics and male reproductive health, such as stem cell therapy showing promise for diabetic erectile dysfunction (51). Reduced testosterone levels are closely associated with dysglycemia (prediabetes and T2DM), and testosterone replacement combined with lifestyle intervention may improve metabolism and sexual function, though further studies are needed to determine whether it prevents progression to overt diabetes (52). In our study, the modest mediation proportion for T2DM (3.80%) indicates that testosterone represents only one of several pathways linking adiposity to diabetes. Future studies should directly measure free testosterone and adopt prospective, sex−stratified designs to validate our findings.

### Strengths and limitations of this study

Integrating observational data with MR, this study explored the association between BFP and DM and found suggestive evidence that increased BFP may increase DM risk via reduced testosterone levels. Clinical information was collected using the hospital’s electronic medical record system with double data entry, which helped to support the authenticity and reliability of the data. The NHANES data have the advantages of a large sample size and standardized variable measurement, which may enhance the extrapolation of the study findings to the real population. The main advantage of the MR method is that it uses genetic instrumental variables to potentially reduce confounding bias, and a variety of statistical methods and sensitivity analyses are supplemented to enhance the robustness of the results.

However, this study has several limitations. (1) Clinical data were collected from patients in the nephrology department, whose metabolic and hormonal profiles may differ from those of the general population. Therefore, these patients cannot fully represent the general diabetic population, and the resulting selection bias may affect the generalizability of the findings. (2) The cross-sectional nature of NHANES data cannot strictly establish the temporal sequence of variables. (3) In the stratified analysis of menopause, the sample size of each subgroup is relatively small, and the statistical efficacy may be insufficient, resulting in the inability to detect a smaller true mediating effect. (4) Residual confounding caused by lifestyle factors (physical activity, diet, medication adherence, etc.) or other metabolic variables cannot be completely excluded. (5) Although a series of sensitivity analyses were performed in the MR analysis, it may still be potentially disturbed by the pleiotropy of genetic instrumental variables, i.e., SNPs may affect DM risk through other pathways independent of testosterone. This limitation is important because pleiotropy remains a key challenge in MR−based analyses. Residual or undetected horizontal pleiotropy cannot be entirely excluded, even after applying detection and correction methods such as MR−PRESSO. (6) The GWAS summary data relied on by the MR study are mainly from European ancestry populations, which limits the generalizability of the causal conclusions to other ethnic groups. (7) The analysis was mainly based on total testosterone concentrations, without fully adjusting for SHBG or evaluating free testosterone, which may affect the measurement accuracy of hormone exposure. In light of the above limitations, future studies should enroll community−based healthy populations from diverse ethnic backgrounds, adopt prospective cohort designs to establish temporal sequences, and directly measure free testosterone to improve exposure assessment. Such multi−ethnic investigations are essential to evaluate the generalizability of our findings across different populations and to explore potential effect modifications by genetic and environmental factors.

## Conclusions

By integrating observational data (clinical data and NHANES) with MR analysis, this study explored the association between BFP and DM, and revealed that testosterone has a significant partial mediating role in the relationship between BFP and T2DM risk, with this effect having obvious gender specificity. This finding suggests that maintaining normal testosterone levels, especially in obese males with testosterone deficiency, may become a potential intervention target for DM prevention. Future studies are needed to further verify these findings in different populations and to deeply explore their molecular mechanisms and clinical transformation value with the help of multi-omics technologies and interventional trials, including a thorough assessment of the long-term safety and risk-benefit ratio of testosterone supplementation.

## Data Availability

The original contributions presented in the study are included in the article/**Supplementary Material**. Further inquiries can be directed to the corresponding author.

## Supplementary material

Supplementary Figure 1 Data screening flow chart. **(A)** Data screening process of Chinese clinical data. **(B)** Data screening process of NHANES data.

Supplementary Figure 2 Scatter plot of the Mendelian randomization analysis. **(A)** Effect of BFP on glucose levels. **(B)** Effect of BFP on T1DM. **(C)** Effect of BFP on T2DM. **(D)** Reverse-direction analysis of fasting glucose levels on BFP. **(E)** Reverse-direction analysis of T1DM on BFP. **(F)** Reverse-direction analysis of T2DM on BFP. **(G)** Effect of testosterone levels on glucose levels. **(H)** Effect of testosterone levels on T1DM. **(I)** Effect of testosterone levels on T2DM. **(J)** Effect of BFP on testosterone levels.

Supplementary Figure 3 The leave-one-out sensitivity analysis of BFP on glucose levels.

Supplementary Figure 4 The leave-one-out sensitivity analysis of BFP on T1DM.

Supplementary Figure 5 The leave-one-out sensitivity analysis of BFP on T2DM.

Supplementary Figure 6 The leave-one-out sensitivity analysis of glucose levels on BFP.

Supplementary Figure 7 The leave-one-out sensitivity analysis of T1DM on BFP.

Supplementary Figure 8 The leave-one-out sensitivity analysis of T2DM on BFP.

Supplementary Figure 9 The leave-one-out sensitivity analysis of BFP on testosterone levels.

Supplementary Figure 10 The leave-one-out sensitivity analysis of testosterone on glucose levels.

Supplementary Figure 11 The leave-one-out sensitivity analysis of testosterone levels on T1DM.

Supplementary Figure 12 The leave-one-out sensitivity analysis of testosterone levels on T2DM.

Supplementary Table 1 Details of instrumental variables used in the Mendelian randomization.

Supplementary Table 2 Logistic regression analysis of the association between BFP and DM risk.

Supplementary Table 3 Linear model diagnosis results in NHANES.

Supplementary Table 4 Mediation analysis in the overall NHANES population.

Supplementary Table 5 Results of the mediation analysis in the female (NHANES).

Supplementary Table 6 Results of the mediation analysis in the male (NHANES).

Supplementary Table 7 Mediation analysis of the Menopausal status subgroup in NHANES.

Supplementary Table 8 Sensitivity analysis of bootstrap resampling number (R = 500/R = 200).

Supplementary Table 9 The detailed parameters of the Mendelian randomization effect.

Supplementary Table 10 Mendelian randomization analysis between T2DM and BFP using instrumental variables selected at varying thresholds.

## References

[1] Collaborators GBDD . Global, regional, and national burden of diabetes from 1990 to 2021, with projections of prevalence to 2050: a systematic analysis for the Global Burden of Disease Study 2021. Lancet. (2023) 402:203–34. doi: 10.1016/S0140-6736(23)01301-6 PMC10364581 37356446

[2] Zhang S Jiang H Wang L Jia X Zhang J Wang H . Longitudinal relationship between body fat percentage and risk of type 2 diabetes in Chinese adults: Evidence from the China Health and Nutrition Survey. Front Public Health. (2022) 10:1032130. doi: 10.3389/fpubh.2022.1032130 36523583 PMC9744757

[3] Elhabashy SA Abdelhaleem BA Madkour SS Kamal CM Salah NY . Body composition and regional adiposity in adolescents with type 1 diabetes: Relation to insulin resistance, glycaemic control and vascular complications. Diabetes Metab Res Rev. (2025) 41:e70041. doi: 10.1002/dmrr.70041 40183230

[4] Kelly DM Jones TH . Testosterone and obesity. Obes Rev. (2015) 16:581–606. doi: 10.1111/obr.12282 25982085

[5] Muir CA Wittert GA Handelsman DJ . Approach to the patient: Low testosterone concentrations in men with obesity. J Clin Endocrinol Metab. (2025) 110:e3125–30. doi: 10.1210/clinem/dgaf137 40052430 PMC12342380

[6] Atlantis E Fahey P Martin S O'Loughlin P Taylor AW Adams RJ . Predictive value of serum testosterone for type 2 diabetes risk assessment in men. BMC Endocr Disord. (2016) 16:26. doi: 10.1186/s12902-016-0109-7 27230668 PMC4882776

[7] Dhindsa S Miller MG McWhirter CL Mager DE Ghanim H Chaudhuri A . Testosterone concentrations in diabetic and nondiabetic obese men. Diabetes Care. (2010) 33:1186–92. doi: 10.2337/dc09-1649 20200299 PMC2875421

[8] Wittert G Grossmann M . Obesity, type 2 diabetes, and testosterone in ageing men. Rev Endocr Metab Disord. (2022) 23:1233–42. doi: 10.1007/s11154-022-09746-5 35834069 PMC9789005

[9] Kapoor D Goodwin E Channer KS Jones TH . Testosterone replacement therapy improves insulin resistance, glycaemic control, visceral adiposity and hypercholesterolaemia in hypogonadal men with type 2 diabetes. Eur J Endocrinol. (2006) 154:899–906. doi: 10.1530/eje.1.02166 16728551

[10] Ahmed F Hetty S Laterveer R Surucu EB Mathioudaki A Hornbrinck E . Altered expression of aromatase and estrogen receptors in adipose tissue from men with obesity or type 2 diabetes. J Clin Endocrinol Metab. (2025) 110:e3410–24. doi: 10.1210/clinem/dgaf038 39833659 PMC12448628

[11] Mohamad NV Wong SK Wan Hasan WN Jolly JJ Nur-Farhana MF Ima-Nirwana S . The relationship between circulating testosterone and inflammatory cytokines in men. Aging Male. (2019) 22:129–40. doi: 10.1080/13685538.2018.1482487 29925283

[12] Rao PM Kelly DM Jones TH . Testosterone and insulin resistance in the metabolic syndrome and T2DM in men. Nat Rev Endocrinol. (2013) 9:479–93. doi: 10.1038/nrendo.2013.122 23797822

[13] Deurenberg P Weststrate JA Seidell JC . Body mass index as a measure of body fatness: age- and sex-specific prediction formulas. Br J Nutr. (1991) 65:105–14. doi: 10.1079/bjn19910073 2043597

[14] Levey AS Stevens LA Schmid CH Zhang YL Castro AF 3rd Feldman HI . A new equation to estimate glomerular filtration rate. Ann Intern Med. (2009) 150:604–12. doi: 10.7326/0003-4819-150-9-200905050-00006 19414839 PMC2763564

[15] Zhang Q Xiao S Jiao X Shen Y . The triglyceride-glucose index is a predictor for cardiovascular and all-cause mortality in CVD patients with diabetes or pre-diabetes: evidence from NHANES 2001-2018. Cardiovasc Diabetol. (2023) 22:279. doi: 10.1186/s12933-023-02030-z 37848879 PMC10583314

[16] An S Ren S Ma J Zhang Y . Association of depression with age at natural menopause: A cross-sectional analysis with NHANES data. Int J Womens Health. (2025) 17:211–20. doi: 10.2147/IJWH.S504748 PMC11794376 39911359

[17] Falk CF Vogel TA Hammami S Miocevic M . Multilevel mediation analysis in R: A comparison of bootstrap and Bayesian approaches. Behav Res Methods. (2024) 56:750–64. doi: 10.31234/osf.io/ync34 36814007

[18] Larsson SC Butterworth AS Burgess S . Mendelian randomization for cardiovascular diseases: principles and applications. Eur Heart J. (2023) 44:4913–24. doi: 10.1093/eurheartj/ehad736 37935836 PMC10719501

[19] Qiu S Liu Z Jiang WD Sun JH Liu ZQ Sun XD . Diabetes and aortic dissection: unraveling the role of 3-hydroxybutyrate through mendelian randomization. Cardiovasc Diabetol. (2024) 23:159. doi: 10.1186/s12933-024-02266-3 38715052 PMC11077732

[20] Tsutsumi T Tsuchiya K . Testosterone and obesity in an aging society. Biomolecules. (2025) 15:1521. doi: 10.3390/biom15111521 41301438 PMC12650755

[21] Perakakis N Farr OM Mantzoros CS . Leptin in leanness and obesity: JACC state-of-the-art review. J Am Coll Cardiol. (2021) 77:745–60. doi: 10.1016/j.jacc.2020.11.069 PMC8483570 33573745

[22] Genchi VA Rossi E Lauriola C D'Oria R Palma G Borrelli A . Adipose tissue dysfunction and obesity-related male hypogonadism. Int J Mol Sci. (2022) 23:8194. doi: 10.3390/ijms23158194 35897769 PMC9330735

[23] Obradovic M Sudar-Milovanovic E Soskic S Essack M Arya S Stewart AJ . Leptin and obesity: Role and clinical implication. Front Endocrinol (Lausanne). (2021) 12:585887. doi: 10.3389/fendo.2021.585887 34084149 PMC8167040

[24] Landry DA Sormany F Hache J Roumaud P Martin LJ . Steroidogenic genes expressions are repressed by high levels of leptin and the JAK/STAT signaling pathway in MA-10 Leydig cells. Mol Cell Biochem. (2017) 433:79–95. doi: 10.1007/s11010-017-3017-x 28343310

[25] Lee AA Den Hartigh LJ . Metabolic impact of endogenously produced estrogens by adipose tissue in females and males across the lifespan. Front Endocrinol (Lausanne). (2025) 16:1682231. doi: 10.3389/fendo.2025.1682231 41180177 PMC12575157

[26] Coppack SW . Pro-inflammatory cytokines and adipose tissue. Proc Nutr Soc. (2001) 60:349–56. doi: 10.1079/pns2001110 11681809

[27] Leisegang K Henkel R . The *in vitro* modulation of steroidogenesis by inflammatory cytokines and insulin in TM3 Leydig cells. Reprod Biol Endocrinol. (2018) 16:26. doi: 10.1186/s12958-018-0341-2 29566712 PMC5863825

[28] Gianatti EJ Grossmann M . Testosterone deficiency in men with Type 2 diabetes: pathophysiology and treatment. Diabetes Med. (2020) 37:174–86. doi: 10.1111/dme.13977 31006133

[29] Zitzmann M . Testosterone deficiency, insulin resistance and the metabolic syndrome. Nat Rev Endocrinol. (2009) 5:673–81. doi: 10.1038/nrendo.2009.212 19859074

[30] Howard EE Shankaran M Evans WJ Berryman CE Margolis LM Lieberman HR . Effects of testosterone on mixed-muscle protein synthesis and proteome dynamics during energy deficit. J Clin Endocrinol Metab. (2022) 107:e3254–63. doi: 10.1210/clinem/dgac295 35532889

[31] Wolfe R Ferrando A Sheffield-Moore M Urban R . Testosterone and muscle protein metabolism. Mayo Clin Proc. (2000) 75:S55–9. doi: 10.1016/s0025-6196(19)30644-5 10959218

[32] Dimitriadis GD Maratou E Kountouri A Board M Lambadiari V . Regulation of postabsorptive and postprandial glucose metabolism by insulin-dependent and insulin-independent mechanisms: An integrative approach. Nutrients. (2021) 13:159. doi: 10.3390/nu13010159 33419065 PMC7825450

[33] Ding EL Song Y Malik VS Liu S . Sex differences of endogenous sex hormones and risk of type 2 diabetes: a systematic review and meta-analysis. JAMA. (2006) 295:1288–99. doi: 10.1001/jama.295.11.1288 16537739

[34] Ruth KS Day FR Tyrrell J Thompson DJ Wood AR Mahajan A . Using human genetics to understand the disease impacts of testosterone in men and women. Nat Med. (2020) 26:252–8. doi: 10.1038/s41591-020-0751-5 32042192 PMC7025895

[35] Ma H Sun J Wu X Mao J Han Q . Percent body fat was negatively correlated with testosterone levels in male. PloS One. (2024) 19:e0294567. doi: 10.1371/journal.pone.0294567 38170701 PMC10763932

[36] Critchlow AJ Alexander SE Hiam DS Ferrucci L Scott D Lamon S . Associations between female sex hormones and skeletal muscle ageing: The Baltimore Longitudinal Study of Aging. J Cachexia Sarcopenia Muscle. (2025) 16:e13786. doi: 10.1002/jcsm.13786 40296368 PMC12037696

[37] Liang G Song Y Wang X Li J Shi H Zhu Q . Serum sex hormone-binding globulin is associated with symptomatic late-onset hypogonadism in aging rural males: a community-based study. Sex Health. (2021) 18:156–61. doi: 10.1071/sh20201 33715769

[38] Grasa MDM Gulfo J Camps N Alcala R Monserrat L Moreno-Navarrete JM . Modulation of SHBG binding to testosterone and estradiol by sex and morbid obesity. Eur J Endocrinol. (2017) 176:393–404. doi: 10.1530/eje-16-0834 28077498

[39] Xu W Qadir MMF Nasteska D Mota de Sa P Gorvin CM Blandino-Rosano M . Architecture of androgen receptor pathways amplifying glucagon-like peptide-1 insulinotropic action in male pancreatic beta cells. Cell Rep. (2023) 42:112529. doi: 10.1016/j.celrep.2023.112529 37200193 PMC10312392

[40] Kooptiwut S Hanchang W Semprasert N Junking M Limjindaporn T Yenchitsomanus PT . Testosterone reduces AGTR1 expression to prevent beta-cell and islet apoptosis from glucotoxicity. J Endocrinol. (2015) 224:215–24. doi: 10.1530/joe-14-0397 25512346

[41] Xu W Morford J Mauvais-Jarvis F . Emerging role of testosterone in pancreatic beta cell function and insulin secretion. J Endocrinol. (2019) 240:R97–R105. doi: 10.1530/joe-18-0573 30601759 PMC6602868

[42] Ko SH Jung Y . Energy metabolism changes and dysregulated lipid metabolism in postmenopausal women. Nutrients. (2021) 13:4556. doi: 10.3390/nu13124556 34960109 PMC8704126

[43] Hirschberg AL . Approach to investigation of hyperandrogenism in a postmenopausal woman. J Clin Endocrinol Metab. (2023) 108:1243–53. doi: 10.1210/clinem/dgac673 36409990 PMC10099172

[44] Henriques FL Buckle I Forbes JM . Type 1 diabetes mellitus prevention: present and future. Nat Rev Endocrinol. (2025) 21:608–22. doi: 10.1038/s41574-025-01128-6 40527975

[45] Eizirik DL Pasquali L Cnop M . Pancreatic beta-cells in type 1 and type 2 diabetes mellitus: different pathways to failure. Nat Rev Endocrinol. (2020) 16:349–62. doi: 10.1038/s41574-020-0355-7 32398822

[46] Hackett G . Type 2 diabetes and testosterone therapy. World J Mens Health. (2019) 37:31–44. doi: 10.5534/wjmh.180027 30079639 PMC6305869

[47] Al-Sulaiti H Diboun I Agha MV Mohamed FFS Atkin S Domling AS . Metabolic signature of obesity-associated insulin resistance and type 2 diabetes. J Transl Med. (2019) 17:348. doi: 10.1186/s12967-019-2096-8 31640727 PMC6805293

[48] Odutola PO Olorunyomi PO Olatawura OO Olorunyomi I . Effectiveness of remote glucose monitoring versus conventional care in diabetes management: A systematic review and meta‐analysis. Med Adv. (2025) 3:28–36. doi: 10.1002/med4.70008 41531421

[49] Gagliano-Juca T Basaria S . Testosterone replacement therapy and cardiovascular risk. Nat Rev Cardiol. (2019) 16:555–74. doi: 10.1038/s41569-019-0211-4 31123340

[50] Zhao JV Schooling CM . The role of testosterone in chronic kidney disease and kidney function in men and women: a bi-directional Mendelian randomization study in the UK Biobank. BMC Med. (2020) 18:122. doi: 10.1186/s12916-020-01594-x 32493397 PMC7271464

[51] Kang J Song Y Zhang Z Wang S Lu Y Liu X . Identification of key microRNAs in diabetes mellitus erectile dysfunction rats with stem cell therapy by bioinformatic analysis of deep sequencing data. World J Mens Health. (2022) 40:663–77. doi: 10.5534/wjmh.210147 35021304 PMC9482859

[52] Corona G Vena W Pizzocaro A Vignozzi L Sforza A Maggi M . Testosterone therapy in diabetes and pre-diabetes. Andrology. (2023) 11:204–14. doi: 10.1111/andr.13367 36542412

